# Methods for Structuring Scientific Knowledge from Many Areas Related to Aging Research

**DOI:** 10.1371/journal.pone.0022597

**Published:** 2011-07-22

**Authors:** Alex Zhavoronkov, Charles R. Cantor

**Affiliations:** 1 The Russian State Medical University, Moscow, Russian Federation; 2 The Biogerontology Research Foundation, Reading, United Kingdom; 3 Department of Biomedical Engineering, Boston University, Boston, Massachusetts, United States of America; 4 Department of Physiology and Biophysics, University of California Irvine, Irvine, California, United States of America; Virginia Tech, United States of America

## Abstract

Aging and age-related disease represents a substantial quantity of current natural, social and behavioral science research efforts. Presently, no centralized system exists for tracking aging research projects across numerous research disciplines. The multidisciplinary nature of this research complicates the understanding of underlying project categories, the establishment of project relations, and the development of a unified project classification scheme. We have developed a highly visual database, the International Aging Research Portfolio (IARP), available at AgingPortfolio.org to address this issue. The database integrates information on research grants, peer-reviewed publications, and issued patent applications from multiple sources. Additionally, the database uses flexible project classification mechanisms and tools for analyzing project associations and trends. This system enables scientists to search the centralized project database, to classify and categorize aging projects, and to analyze the funding aspects across multiple research disciplines. The IARP is designed to provide improved allocation and prioritization of scarce research funding, to reduce project overlap and improve scientific collaboration thereby accelerating scientific and medical progress in a rapidly growing area of research. Grant applications often precede publications and some grants do not result in publications, thus, this system provides utility to investigate an earlier and broader view on research activity in many research disciplines. This project is a first attempt to provide a centralized database system for research grants and to categorize aging research projects into multiple subcategories utilizing both advanced machine algorithms and a hierarchical environment for scientific collaboration.

## Introduction

Aging is a multidisciplinary, multi-billion dollar field covering a broad range of natural, behavioral and social science research disciplines. Despite decades of research into understanding the causes, mechanisms, behavioral and social aspects of aging, it remains one of the most complex, controversial and widely disputed areas of science. Presently, many biomedical research tools exist to search, analyze and categorize data in the form of articles, patents, clinical trials, project descriptions and grants. However, there are also a substantial number of projects supported by governmental, profit and non-profit funding that are not covered by PubMed and other popular scientific resource platforms. The International Aging Research Portfolio (IARP) is intended to provide access to information on these research activities to allow for better planning and decision-making within universities, research organizations, funding agencies and laboratories. Moreover, it aims to improve scientific collaboration, research performance and innovation capacity among the research community.

### Limits of classical literature search

**Figure 1:**
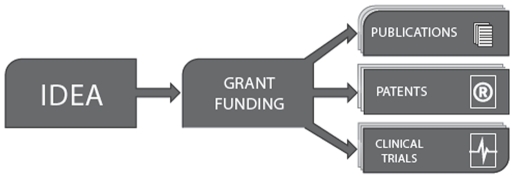
General scheme of scientific research. The scheme illustrates the general concept where research grants precede publications. It allows for the possibility for grant applications to be published earlier than research results and failed research projects receiving grant funding not to be published at all.

The volume of biomedical literature grows at a remarkable pace. PubMed, the main biomedical literature database, currently references more than 15,000,000 abstracts. Owing to its size, a simple keyword search of the literature often does not yield relevant target-specific information, and therefore important information often remains inaccessible in the mass of text. Search engines have been developed for classification and advanced investigation of publications, such as GoPubmed, a hierarchically structured vocabulary for molecular biology which explores PubMed search results using Gene Ontology (GO) [1]. These advanced search engines have become very useful for classifying and structuring research that would not otherwise be easily retrievable via resources such as PubMed. However, even these advanced engines neglect highly relevant information that is contained in grant applications, reflecting the most recent funding trends and research ideas **(** **Figure 1** **)**. Grant applications may often precede a publication or patent by several years. If there are issues with experimental procedures and/or process the investigations may omit these details in subsequent grant applications or publications. For many research initiatives, publications constitute only part of the relevant project results, with the remaining data not being published until patent approval or the start of clinical trials. Therefore, with access to a centralized database, researchers may view recent research activity by searching or browsing through a directory of grant applications in their respective fields of research in the IARP.

### Timing and project tracking

Most contemporary projects are funded by government, non-profit, or commercial organizations, and many years may elapse between the time the project is funded and when the results are published in peer-reviewed literature or a patent application. Consequently, there is a need for an international inter-organizational system for tracking grants to avoid funding overlap and to improve cooperation between government, non-profit and commercial entities. The IARP is designed to track publications, clinical trials and patents that are linked to grants. It also identifies related and similar projects around the world using an algorithm analysis of project abstracts, patents, and publications linked to the project. This type of advanced project analysis will potentially aid in circumventing project overlap and redundant funding of similar programs while also highlighting knowledge gaps for potential funding.

### Current project databases

There are multiple widely-used databases for tracking and categorizing research projects; however, there is currently no comprehensive resource for the detailed tracking of projects specifically related to aging research. The global scientific community actively uses numerous systems of this kind, such as CancerPortfolio, which was developed for tracking cancer research and classifying the research into different categories. Another well-known database is ClinicalTrials (www.clinicaltrials.gov), which contains information on federally and privately supported clinical trials conducted in the United States and around the world. ClinicalTrials provides information about the purpose of a trial, its participants, locations and phone numbers [2]. Novoseek is a search engine with classification of publications and grants using most frequent categories as keywords [3]. Although Novoseek is also widely used by researchers, it does not provide flexible graphic representations of the data the user retrieves. Moreover, a serious limitation of resources like CancerPortfolio (www.cancerportfolio.org), Novoseek, and ClinicalTrials is that they do not contain comprehensive information on international research project funding, which is a very important datapoint and can be used to analyze the size, scope and length of the project.

Within a similar theme, HeathCompetence.eu (www.healthcompetence.eu), includes all projects related to health and the life sciences supported by European Commission framework programs since 2004, and has several categories primarily for diseases without any hierarchical structure. Additionally, CORDIS (www.cordis.europa.eu) a well-known European Commission portal on framework programs includes information on all EU projects with historical data in different areas of research from physics and medicine to agriculture and aerospace. Other web-based databases include the Canadian Institutes of Health Research Database (www.researchnet-recherchenet.ca) and the Australian National Health and Medical Research Council Database (www.nhmrc.gov.au), which contains a search engine and table view of information related to health projects supported by the government. The NIH RePORTER (report.nih.gov) is a web-based search engine of intramural and extramural National Institutes of Health-funded projects, categorized by experts into 218 research areas, although mostly disease-related research. The NIH RePORTER enables the user to view funded projects on the map and provides several graphical views based on query results of interest. However, such databases do not have any hierarchical categorization of projects making it difficult to find projects related to different aspects of aging research.

When designing the IARP, we analyzed the most popular features from the systems described above while focusing on providing maximum flexibility. It was our aim to create a robust system wherein a cogent categorizing process and funding data for each project would identify areas receiving the greatest funding and areas that may be in need of increased funding. We also linked the projects to related publications in the MEDLINE database. The system has sophisticated modules of reporting tools that enable comprehensive reports to be generated for individual organizations and researchers, benchmarking per thematic area, as well as collaboration analysis on selected partner organizations and regions. Researchers can also become category editors for the IARP system allowing them to log access the system to retrieve, add, validate, and use data on projects, publications and patents while also being able to map projects to research categories. Additionally, Scientific Advisory Board (SAB) members will have access to additional functionality, for example, to add their own categorization taxonomies, and to add or to decline category editors.

## Results and Discussion

We have developed an IARP to facilitate a better understanding of the field of aging research and provide a more structured view on this complex multidisciplinary research area. Currently, the database integrates all available funding data on aging and age-related research projects financed by National Institutes of Health, European Commission, and National Research Council Canada, averaging approximately 10 years of historical funding data (1991–2010 for NIH, 2000–2010 for European Commission, 2008–2010 for National Research Council Canada). The database also includes a copy of the MEDLINE abstract database with many research projects linked to resulting publications. The database also includes developed semantic classification algorithms to classify projects automatically into research areas specifically related to aging. The flexibility of this system provides an insight into aging and age-related projects to identify research funding trends and to analyze the current status of the project. It also gives users the opportunity to build new classification taxonomies to identify issues associated with aging research from different perspectives.

### Peer-reviewed Manual Project Categorization Mechanisms

**Figure 2:**
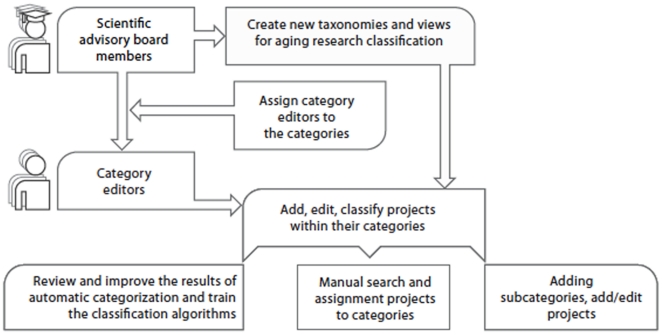
Project management scheme for category editors and SAB members. The two main user classes are the scientific advisory board (SAB) members and category editors (CE). SAB members can assign category editors so specific categories. Both user classes can add, edit, classify the projects, add or edit subcategories and train the classification algorithms.

As current classification algorithms are limited and custom classification mechanisms for aging research are needed, this system also allows for the development of broad scientific advisory and category editorial boards to add, edit and classify projects manually. The best practices for the hierarchical category editor structure were taken from the Netscape Open Directory Project, the largest human-edited Internet directory used by Google Directory (www.dmoz.org, with over 590,000 categories and 85,000 human editors), allowing SAB members, institute directors and laboratory heads to supervise high-level categories and delegate the management of lower-level categories to category experts, graduate students and subordinates. The scheme of project management is presented in **Figure 2**. The system currently allows three main types of users:

- *Administrators* – technical experts responsible for system development and maintenance.
- *SAB members* – established aging and aging-related disease scientists with a large number of publications in peer-reviewed journals. The SAB members can be invited by the chairman of the SAB, by other SAB members, or they can register manually online. All new SAB members must be approved by the SAB chairman and at least five other SAB members. The SAB members are responsible for assigning top category editors and they can create and manage their own category taxonomies and manage projects within their own views of theories of aging.
- *Category editors* – experts in their respective categories or category groups responsible for editing, adding and linking projects to the default taxonomies. Every category editor and SAB member can add keywords for their categories to facilitate searching projects and manage automatic categorization results by approving or declining projects for each category.

### Advanced tools for group project management

The back-end part of the system contains tools for project categorization available only for registered category editors and SAB members. The category management tools available to SAB members allow for adding/editing the category taxonomies and adding new views on aging research classification. The category management tools are available both to SAB members and category editors. These allow users to add/edit projects; search projects by category or keywords in the title of a project abstract and description; and manage categories for every project in the table of search results. The system shows the relevance for each category established using the Support Vector Machine (SVM) algorithm.

### Open Architecture and the Roadmap for System Development

Currently, the system contains the grant databases from the NIH, the European Commission and several other funding bodies mapped into the highly-scalable, easy-to-use database structure. It also contains the MEDLINE abstract database and links to related publications, clinical trials and patents. It may be possible to use this vast amount of structured textual data to develop an Application Programming Interface (API) for representing and visualizing and/or enhancing the biological pathways. In a planned system extension, a user can upload a regulatory network of interacting proteins or genes with filtering opportunities, for example, visualizing a regulatory network from the point of breast cancer, the user will assign the keyword “breast cancer” to the pathway. The system will automatically build the network with nodes reflecting proteins or genes in that network, where size of node represents the number of projects, publications and funding, and edges reflects biological interaction. Such a tool will allow the investigator to look at the systems biology level of funding and help find knowledge gaps in the research.

### Conclusion

We have developed a knowledge management system for aging research projects, the International Aging Research Portfolio, to facilitate a better understanding of the field of aging research and the organization of initiatives within. This system contains a compiled database of projects funded by the NIH and the European Commission and uses semantic classification algorithms to automatically classify projects into research areas related to aging. The flexibility of the system provides different views on aging and age-related projects in order to find trends and analyze the current status of the investigations. It also provides the user the opportunity to build new classification taxonomies to look at the problem of aging research at different points. This centralized knowledge management system for tracking such a complex, interdisciplinary and controversial area as aging research is available to the public via the AgingPortfolio.org website.

## Materials and Methods

### Automatic Project Classification

To identify projects related to aging research within a large dataset and to separate the projects into relevant semantic groups, we have developed a system for automatic project classification. The system utilizes two classification algorithms with elements of machine learning: SVM and Recurrent-Neural-Network-Based Boolean Factor Analysis (BFA).

### Support Vector Machine (SVM)

Problem solving consists of two steps: learning and prediction. A SVM algorithm is used for the learning step. This method builds on the basis of a training set hyperplane which separates vectors in n-dimensional space. Unlike other methods, SVM constructs an optimal separating hyperplane in the sense that bandwidth dividing classes are the maximum. That is, the input is a training sample: pairs <vector, rubric>. The result of the learning process is a rule in the form 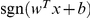 where 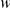 – sought for vector, 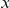
– vector representation of project, 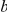 – sought for threshold. SVMs are a new learning method introduced by V. Vapnik et al. [4]. They are well-founded in computational learning theory and are very open to theoretical understanding and analysis. SVMs are based on the Structural Risk Minimization principle [5] from computational learning theory.

One describes the problem to be solved to find the unknown 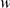 and 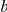. If given n-dimensional training vectors 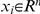, 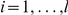, in two classes, and a vector 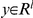 such that 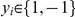 (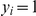 means that the vector 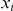 belongs to one category, and 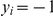 means that the vector 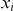 belongs to another category), then SVM solves the following problem, denoted as (a), for constructing an optimal separating hyperplane:
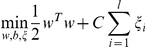
subject to 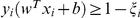


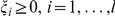




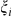
 is additional variable that characterize the magnitude of the error on objects 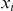 belonging to the training set. 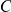 is the parameter of the algorithm (the nature of this parameter is rooted in the method of Tikhonov regularization [6]).

According to the Kuhn-Tucker theorem [7], dual (a) problem is
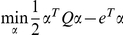
subject to 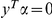




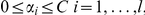
where 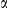 is a dual variable to 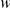, 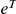 is the vector of all ones, parameter 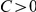 is the upper bound, 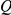 is an 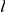 by 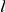 positive semi-definite matrix, 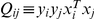. Let's denote this problem (b). To solve problem (b) is to use the module for solving optimization problems TRON [8].

In going from (a) to (b) we obtain the relation 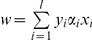. Therefore, the decision function is
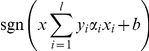
where 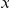 is vector one wants to categorize, 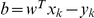, where 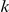 is such that 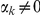.

The decision rule, which was received at the learning step, is used at the prediction step. Hence, prediction is reduced to the multiplication of two vectors: the vector, which was built at the training stage, by the vector one wants to categorize in the prediction step.

To represent a project as a vector the vector space model (or term vector model) is used with weights calculated by the following TF-IDF [9] formula:



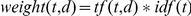





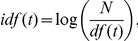
where 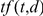 is frequency of the term 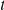 in the document 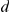, 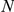 is total number of documents in collection, 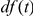 is number of documents to which a term 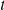 is assigned. The terms are all words from the descriptions and titles of projects in the training set, with the exception of stop words. Each vector is normalized to a vector with a Euclidean norm of 1. Thus, all vectors belong to the n-dimensional sphere of radius 1.

**Figure 3:**
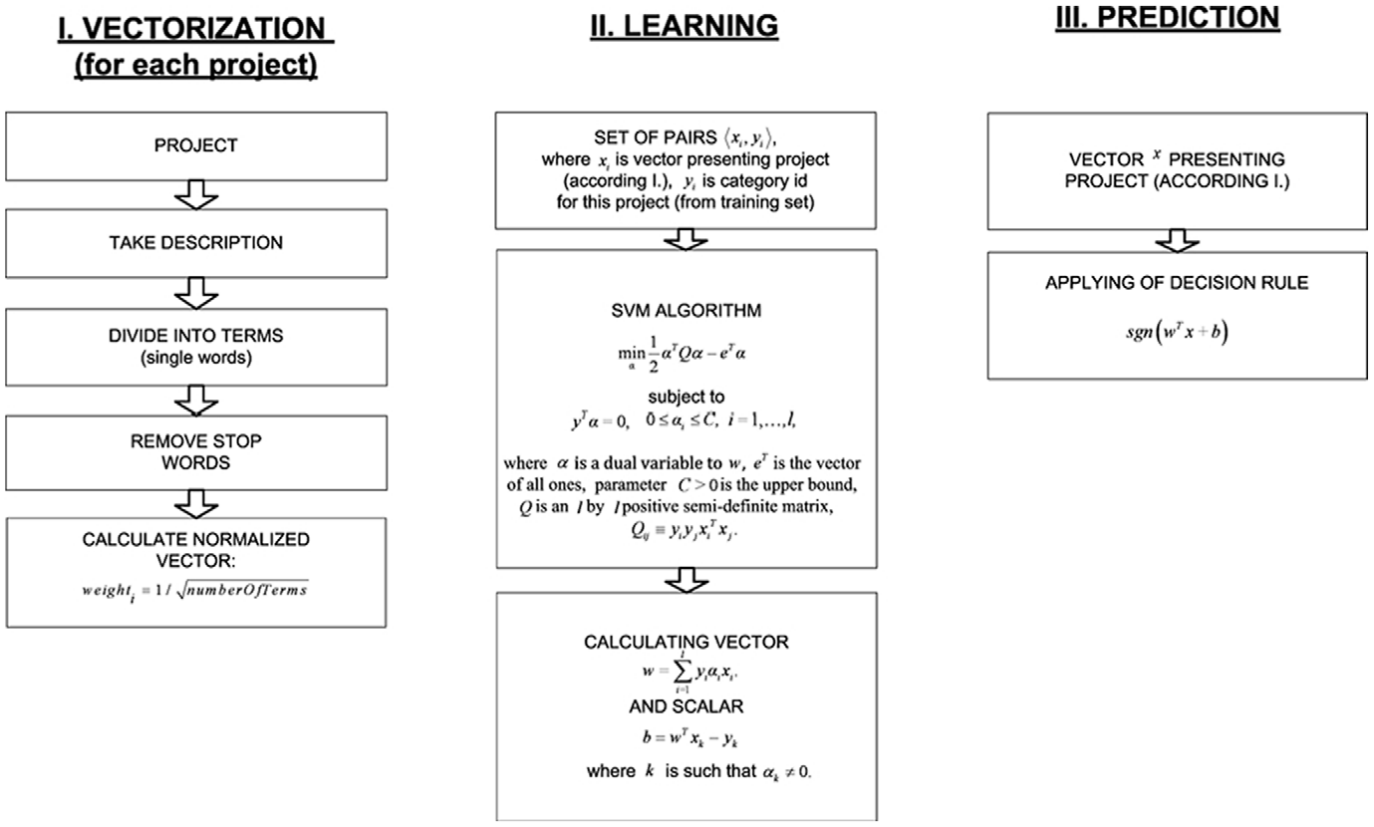
Workflow of the Support Vector Machine (SVM) algorithm implementation. (A) Vectorization step for each project in the database. (B) Learning step using the Support Vector Machine (SVM) algorithm with the training sets. (C) Prediction of project classification.

Thus, as described above, 3 modules are involved in the scheme of the program: vectorization of projects, learning and using the training set, classification of new projects (prediction). Schemes of the sequences of steps for each of these modules are reflected in **Figure 3** **.**

The multiclass problem is reduced to the procedure of training None-vs-the-rest real-valued classifiers to obtain a length-N output vector and testing new examples by predicting the class with the largest binary prediction score.

There are two training sets of projects associated with each category in the taxonomy: “classify” and “do not classify”.

The IARP system incorporates project data from many international sources and the quality, granularity and available project properties are difficult to control. The projects manually included into the training sets include all possible text fields such as the title, abstract, field of tags and associated keywords, principal investigator name, institution, associated publications and many other project properties.

In the IARP system, each category in the taxonomy contains a “classify” training set of minimum 50 projects directly related to the category. Some popular categories have many hundreds of projects in “classify” training sets. These projects were assigned to the categories by the expert category editors or science advisory board members using the administrative interface tools of the system.

These training sets are later used by the classification algorithms to identify simillar projects in the project databases. After each automatic classification, the administrative interface allows category editors to review the projects marked “classified automatically” in their respective categories. After the editor marks the project as classified properly, the project is added to the “classify” training set and is used for automatic classification. In the case that the editor decides that the project is not related to the category, it is added to the “do not classify” set. The editors and science advisory board members have the option to discuss the classification of each individual project in project properties or at the IARP discussion forum. The training sets for categories are expanded on a regular basis and classification accuracy improves over time.

To evaluate the accuracy of the classification algorithms a separate set of projects called the “reference sample” was created by a team of category editors that did not previously participate in the creation of the training sets. The reference sets contain several thousand projects assigned to one or several categories and are expanded on the regular basis. The reference sets and the training sets do not intersect. The results of automatic classification using new revisions of the algorithm are compared to the reference sets to evaluate classifcation accuracy.

### Recurrent-Neural-Network-Based Boolean Factor Analysis (BFA)

The general BFA method is based on the Hopfield-like attractor neural network (ANNEA, Associative Neural Network with Expanding Activity) [10]. This method exploits the well-known property of a Hopfield network to create attractors of network dynamics by tightly connected neurons, representing terms in text analysis. Since neurons representing a category are activated simultaneously each time the category appears in the patterns of the data set, and neurons representing different categories are activated simultaneously only by chance, then via the Hebbian learning rule the neurons (terms) of categories become connected more tightly than other neurons. Hence, the category can be revealed as a property of the network dynamics. This method was demonstrated to be effective for textual data analysis [10]–[12].

#### The IARP taxonomies

To classify and study trends in modern aging research, we have designed classification taxonomy of the main fields of aging studies. These fields were selected by the comparative analysis of projects and publications containing the terms “aging” and “ageing” in the publication databases (Medline (www.ncbi.nlm.nih.gov), N.O. and Biological Abstracts (www.thomsonreuters.com/products_services/science/science_products/a-z/biological_abstracts) for natural studies, as well as Wilson Social Sciences Abstracts (www.hwwilson.com/Databases/socsci.cfm), the American Psychological Association PsycINFO (www.apa.org/pubs/databases/psycinfo/index.aspx) and the American Economic Association EconLit (www.aeaweb.org/econlit/index.php) for social and psychological ageing research). To avoid under-representation of certain terms we conducted a Google Scholar (scholar.google.com) search for aging-related terms. Further term analysis was carried out using the frequency of term occurrence i.e., related terms were selected from the LitMiner tool (andromeda.gsf.de/litminer) and the Zotero research (http://www.zotero.org) for analysis of selected article abstracts.

By analyzing the NIH and the EC grant databases we identified two main classes of projects: natural sciences and behavioral and social sciences. And three auxiliary classes that may be of interest to several user types: infrastructure development, events and training programs, theories of aging.

The main aspects of aging research in sociology, psychology and other related disciplines were formulated by the comparative study of multiple sources. Initially, we took the formulated topics at the official site of the Alliance for Aging Research (www.agingresearch.org/section/topic) and also the classification of the main tasks at Sociosite (www.sociosite.net/topics/aging.php). It should be noted that several aspects of healthy aging, e.g. increased traumatic injury risk for elders, intersect with medical studies of the Natural Sciences component, thus, we have shortened the list of main subcategories of the field of behavioral and social sciences by the thorough analysis of recent publications, including reviews [13]–[23]. Subsequently, second-level categories of behavioral and social sciences were formulated:

- Psychology of Aging
- Healthy Aging/Health promotion
- Population Studies
- Policy research
- Quality of Life/Well-Being
- Physical and Mental Functioning
- Care systems access
- Family Relations/Intergenerational Transfers
- Social Influences and Cognition
- Race and Ethnic Relations

For each category, distinct third-level subcategories were designed using the most frequent terms found in these fields of the behavioral and social sciences branch of aging research (Google Scholar, Social Sciences Abstracts, PsycINFO and EconLit). We have introduced a weighting for each low-level subcategory by the number of related publications (over a 5 year period).

Due to the interdisciplinary character of aging research [24], [25], the lower-level taxonomy for the *Natural sciences* is devoted to the main aspects of aging research. This should lead to the identification of the mechanisms of aging and, possible strategies to increase well-being and vitality in the context of aging and health. Thus, we have divided the category *Natural sciences* into the following sub-categories:

- Aging Diseases & Pathology
- Model Organisms
- Aging Mechanisms by Anatomy
- Clinical Trials and Therapy
- Aging-related Markers and Targets
- Proteins, Genes and Regulatory Networks
- Experimental Techniques
- Metabolism and Nutrition
- Anti-aging Strategies

The mechanisms pertaining to age-related disorders have been studied extensively in human and model organisms [26]–[30]. We have selected medical disease terms based on the standard CDC data list (Centers for Disease Control and Prevention, www.cdc.gov/az/a.html) and the medical disease ontology (see NCBO Bioportal, bioportal.bioontology.org/ontologies/35686). We filtered known diseases and classified them for their focus on aging research on the basis of the following assumptions:

1. Different classes or types of cancers, and cancer-related diseases, occur with varying frequencies in younger and elder patients, thus not all cancer studies are closely related to aging.
2. Aging increases vulnerability to various pathologies, including those associated with decelerating regeneration.
3. Aging frequently leads to multiple chronic diseases.

Most chronic diseases, including multiple cancers, also possess genetic predispositions. Thus, we can consider the onset and clinical course of some age-related diseases as dependent on a set of gene polymorphisms. The population frequency of disorders and their severity were used in selecting key terms for categories. For example, benign tumors are prevalent in the elderly but their impact on lifespan is often negligible [31]–[33]. These disorders were grouped into categories by their cellular origin (fibroma, adenoma, lipoma, etc.) cancers, and cancer-related diseases, were filtered by the age-related statistics [34] (apps.nccd.cdc.gov/uscs/United States Cancer Statistics (USCS)), (hseer.cancer.gov/statfacts/Cancer Stat Fact Sheets). Only those cancer-related diseases and malignancies that occur mostly in the elderly were selected for as subcategories. The analogous selection was carried out for other human diseases. Medline searches were performed for disease terms coinciding with “age”, “aged”, “ageing”, “aging”, “senescen*” and “elder”, but not coinciding with “child”, “young” or “childhood”.

The non-human part of aging research was distinguished by the “NOT human” limit for PubMed search related to aging, and the most frequently used organisms were selected. Their evolutionary relation to humans was established based on the NCBI taxonomy; both primate and rodent studies could be more relevant to human aging, while most current or past studies are based on model organisms such as yeast, fly and nematode. Although the investigation of organisms of different levels of organization can be informative, there are well-known pitfalls of attempting to generalize aging in a model organism.

The cell, tissue, organ, and system levels of aging-related changes were selected as second-level categories for *Aging mechanisms by anatomy*. The main terms were selected based on Science Prof Online (www.scienceprofonline.org/), CellsAlive! (www.cellsalive.com/), InnerBody (www.innerbody.com), Visible Body (www.visiblebody.com) and Medline data sources. Other natural sciences-related aging research projects and publications were manually studied and the second-level categories were formulated. These categories were subdivided by frequency of terms in publication abstracts and significantly related subcategories were merged. For example, we merged aging biomarkers and drug targets into *Aging-related markers and targets*, as aging-related biomarkers were investigated in numerous studies, and some of them were selected as drug targets for anti-cancer or anti-aging therapies [35]–[38]. Currently the system contains two main top-level categories: natural and behavioral and social sciences and three auxiliary top-level categories: events and training programs, theories of aging and infrastructure.

#### Using AgingPortfolio.Org

**Figure 4:**
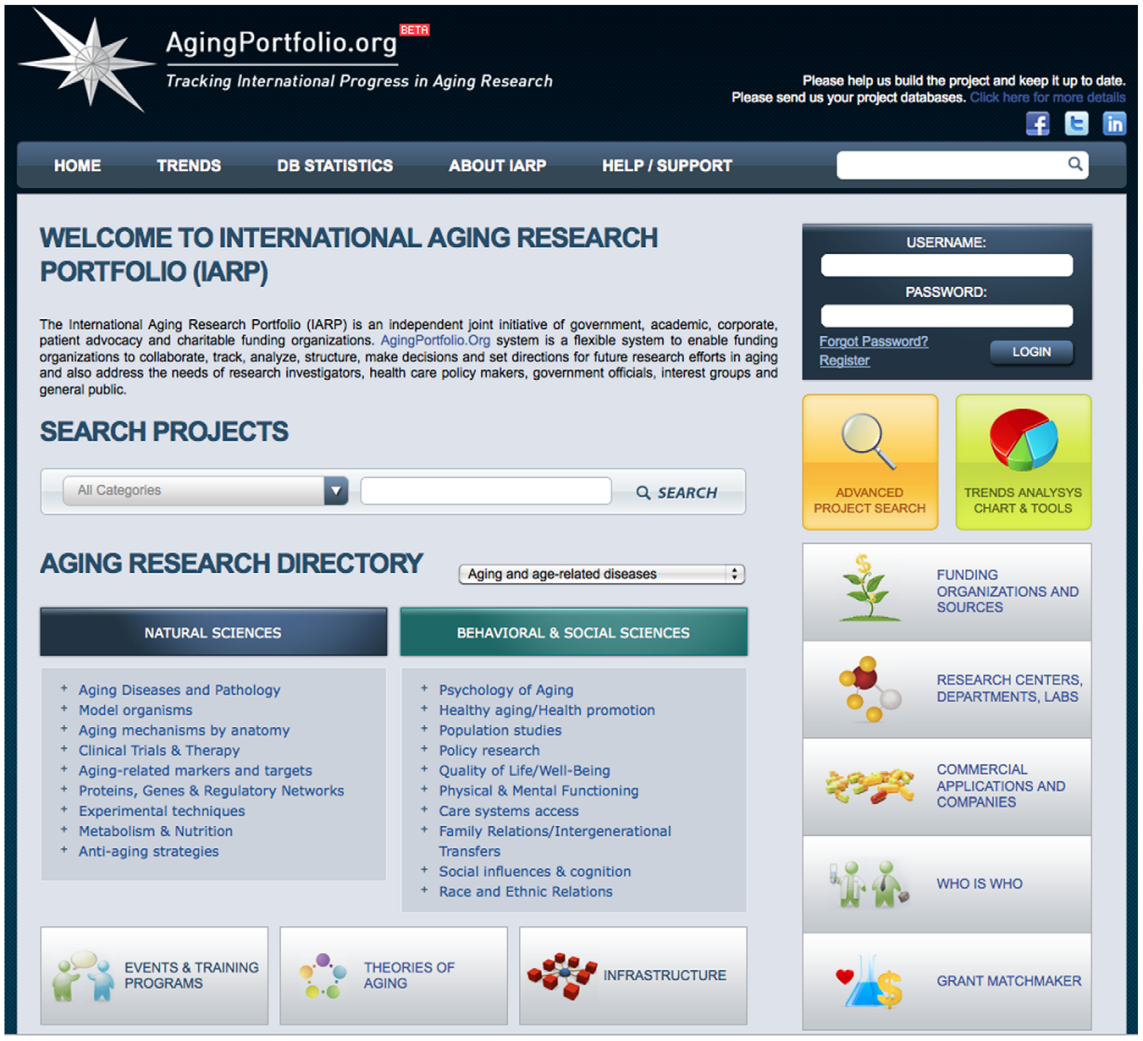
Screenshot of the AgingPortfolio.org web-site home page interface. Screenshot of the website taken on the date of manuscript submission illustrating the main features and the research project classification directory.

**Figure 5:**
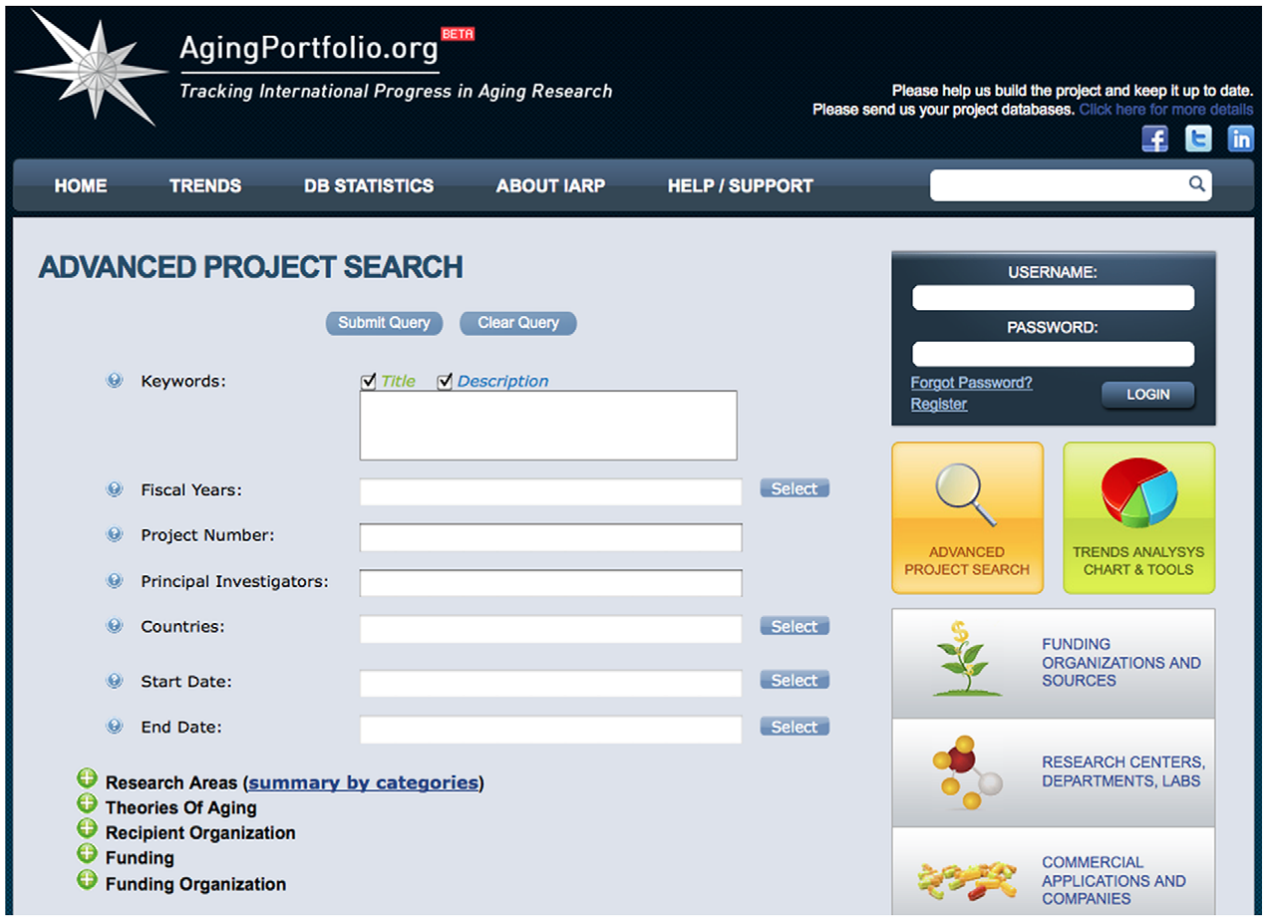
Screenshot of the Advanced project search page. Screenshot of the website taken on the date of manuscript submission illustrating the advanced project search parameters and limits.

The IARP is a flexible system that allows for the easy handling of information, performance of complex queries and data analysis in specific areas of interest. It has a set of tools for simple, rapid and productive searching. The features of the system include time series and trend analysis; funding institution comparisons; identification of project intersections and overlap by research area, condition, disease, an advanced overview of funding flow with top down and bottom up analysis; comparison of organizations by a large number of criteria; browsing of projects by geography; and visualization of number of projects, research dollars, number of investigators, funding organizations, and related projects. The web-site home page consists of three main parts **(** **Figure 4** **)**:

1. The navigation menu at the top of the page provides quick links to the home page, trends described in detail below, database statistics representing a comprehensive analysis of data stored in the system (useful for understanding the content of the database by total number of projects), the top 5 active, completed projects by total funding, the top 5 countries, US states, investigators, funding bodies and recipient organizations, also by total funding. The Help/support section contains information on how to use the system. Additionally, a wiki online collaboration system has been implemented to provide registered system users, developers and collaborators the opportunity to build the knowledge base for the system and to collaborate on system development. The About IARP section contains project description and information on project goals, science advisory board (SAB) members and developers while the quick site search section provides a brief overview of the projects by search criteria.
2. The left side of the website contains the Top-level Categories section, with links to subcategories and a map view with colored countries reflecting the amount of funding in each fiscal year. The IARP is built on a broad theoretical framework; it allows broad classification, consideration of different approaches, theories of aging and analysis of investigations in various regions of the scientific world. The drop down menu allows for multiple views of the taxonomy based on the standard or user-defined limits. The limits feature enables users to narrow down the top-level directory by excluding the infrastructure investments, clinical trials and projects that have lower relevance to the aging research. The Top-level Categories section allows comparison of the contribution of researchers in a variety of areas, assessing the amount of funding going into specific areas of research which provide a more structured representation of the aging research activities. The Theories of Aging groups the research projects into the known theories of aging. The SAB members can suggest new theories to be added to the section and populate it with projects from the database using the automatic project classification algorithms or manual project categorization protocols. Overviews of funding of infrastructure and equipment are also important for experimental and theoretical studies. Contribution of events and training programs in aging should also be taken into account, as this can lead to scientific collaborations and professional development among users.
3. The right side of the website contains various tools and additional search engines: (a) Advanced Project Search **(** **Figure 5** **)** allows the user to choose a variety of criteria, ranging from the date of creation and research and funding mechanism to specific projects. Each project contains not only a description, but specific information about the organizers and researchers. Moreover, it links the current project with other similar projects and financial mechanisms. (b) Trends Analysis Chart & Tools is a collection of tools for visualizing the project data in the form of diagrams, charts and comparative tables. This provides different visual representations of data on financing, depending on the criterion of interest (the institution, the university researcher, region, and category). By using (c) Research Centers, Departments, Labs, (d) Funding Organizations and Sources, and (e) Commercial Financing and Companies, the user can analyze trends in funding by categories, compare different organizations by year of funding, by total number of funded projects and fiscal year. Organizations can also be filtered by year of funding, funding mechanism, category, country, or US state. (f) Who is who identifies key investigators by research area, amount of funding, institutes, companies, universities, not-for-profit and profit organizations, funding bodies and corresponding funding mechanism, country, US state and displays the total amount of funding for all years and provides time series graph view by fiscal year of funding. For most investigators, contact information and work addresses are available.

Through the use of the IARP one can clearly see the state of affairs in areas of aging research, and can also use these findings to make better decisions and develop better research funding policies for future studies.

### Examples

Example 1: A scientist involved in a research project related to nuclear DNA damage in aging and cancer is interested in identifying related active and completed projects, organizations, funding sources and other principal investigators in this area of research.

The IARP system rovides several ways to obtain this data:

Using the default system taxonomy by traversing the following path from the main page: Natural sciences > Aging and Related Diseases > Aging Mechanisms by Anatomy > Cell Level > DNA

Using the project search engine: Advanced project search > Search Terms: “DNA AND damage OR mutations AND cancer”

The scientist can then sort the results by year and find the most recent grants that may not have resulted in pubilcations.

Example 2: A scientist performing experimental research on aging mechanisms of *C. elegans* in his state or country can go into the category Natural sciences > Model Organisms, select the country he is looking for or retrieve the data by a keyword search using “*C. elegans”* or “worm” keywords.

Example 3: A PhD student interested in fellowships on regeneration can go to “Advanced Project Search”, select “Event and Training Programs” and “Bioengineering & Regeneration” categories, and funding mechanism “Fellowships”, and then view the search results as graphs by the top 5 investigators, funding by year, by country, by US state, and recipient organizations.

Example 4: An investigator who is interested in a particular category can view top funding organizations, top investigators, countries, US states and funding mechanisms by funding. The system also allows one to find the most relevant and related projects to the one selected.

Example 5: A funding organization or institute administration can select different organizations and find out which categories have overlapping projects and funding, and can find other organizations by categories all over the world.

A funding organization may also use the Who is Who tool with “Category” subfilter to browse through principal investigators receiving the most funding from other organizations. This may be especially useful when trying to identify experts for peer review, consultation or collaboration.
